# Management of Primary Refractory Diffuse Large B‐Cell Lymphoma in Patients Unsuitable for CAR T‐Cell Therapy

**DOI:** 10.1111/ejh.70153

**Published:** 2026-03-07

**Authors:** Santino Caserta, Enrica Antonia Martino, Mamdouh Skafi, Maria Eugenia Alvaro, Antonella Bruzzese, Nicola Amodio, Eugenio Lucia, Virginia Olivito, Caterina Labanca, Francesco Mendicino, Ernesto Vigna, Fortunato Morabito, Massimo Gentile

**Affiliations:** ^1^ Department of Onco‐Hematology Hematology Unit, AO of Cosenza Cosenza Italy; ^2^ Emergency and Internal Medicine Department Saint Joseph Hospital East Jerusalem Palestine; ^3^ Department of Experimental and Clinical Medicine University of Catanzaro Catanzaro Italy; ^4^ AIL Sezione di Cosenza Cosenza Italy; ^5^ Department of Pharmacy, Health and Nutritional Science University of Calabria Rende Italy

**Keywords:** antibody–drug conjugates, bispecific antibodies, CAR T‐cell therapy, CAR T‐cell unsuitability, disease progression, DLBCL, primary refractory DLBCL, salvage therapy

## Abstract

Primary refractory Diffuse Large B‐Cell Lymphoma is associated with poor outcomes and limited responsiveness to conventional salvage therapies. Although CAR T‐cell therapy represents the standard of care in this setting, a substantial proportion of patients cannot receive it despite meeting disease‐related criteria. In this review, “unsuitable” refers to patients who are temporarily or functionally unable to undergo CAR T‐cell therapy because of reversible clinical conditions, rapidly progressive disease requiring immediate cytoreduction, or logistical and social barriers, rather than permanent contraindications. For these patients, prompt alternative strategies are required. Conventional platinum‐based or gemcitabine‐ and bendamustine‐containing regimens retain a role for short‐term disease control but offer limited durability. In contrast, novel antibody‐based therapies, including polatuzumab‐containing combinations, loncastuximab tesirine, and tafasitamab plus lenalidomide, have expanded treatment options with improved tolerability. Most notably, CD20 × CD3 bispecific antibodies represent a major therapeutic advance, providing off‐the‐shelf immune engagement with predominantly outpatient administration. From a practical perspective, early identification of reversible barriers to CAR T‐cell therapy and timely use of bispecific antibodies or other antibody‐based regimens are critical to achieve rapid disease control, preserve organ function, and, when feasible, restore eligibility for cellular therapy.

## 1 Introduction

Primary refractory diffuse large B‐cell lymphoma (DLBCL) is among the most challenging aggressive B‐cell malignancies. It is conventionally defined as a disease that fails to achieve a complete remission with standard first‐line chemoimmunotherapy—most commonly R‐CHOP—or that progresses within 3–12 months after treatment completion. This category also includes patients with stable or progressive disease during induction, reflecting resistance to frontline therapy [1]. Biologically, primary refractory DLBCL (PR‐DLBCL) frequently harbors high‐risk molecular features such as activated B‐cell (ABC) phenotype, double‐expressor status, or rearrangements of MYC and BCL2/BCL6, which collectively contribute to early treatment failure and an aggressive clinical behavior [2].

Epidemiologically, primary refractory disease accounts for approximately 10%–15% of newly diagnosed DLBCL cases but contributes disproportionately to lymphoma‐related mortality. Population‐based registries consistently demonstrate inferior survival compared with patients who relapse later or initially respond to therapy. Historically, median overall survival following documentation of refractory disease has been measured in months, with limited benefit from conventional salvage regimens. Outcomes are particularly poor in individuals with high tumor burden, early progression, or adverse biological features, underscoring the need for rapid and effective therapeutic intervention [3].

Over the past decade, chimeric antigen receptor (CAR) T‐cell therapy has transformed the management of relapsed or refractory DLBCL and is now considered the standard of care for eligible patients with primary refractory disease [4, 5]. Pivotal trials demonstrated that CD19‐directed CAR T‐cell products induce durable remissions in a substantial subset of patients, outperforming salvage chemotherapy and autologous stem cell transplantation (ASCT). Consequently, CAR T‐cell therapy is now recommended after first‐line failure in patients with refractory or early‐relapsed disease, representing a paradigm shift away from platinum‐based salvage approaches [4, 5].

Despite these advances, a substantial proportion of patients with PR‐DLBCL cannot proceed to CAR T‐cell therapy due to clinical instability, organ dysfunction, socioeconomic limitations, or logistical barriers. Optimal management of these individuals, therefore, remains an urgent unmet need [6].

This review synthesizes current evidence on the biology, clinical features, and management of PR‐DLBCL in patients unsuitable for CAR T‐cell therapy, focusing on available therapeutic options and emerging strategies.

## 2 Pathobiology and Clinical Features of Primary Refractory DLBCL

Unlike patients who relapse after an initial response, those with primary refractory disease display an intrinsic lack of chemosensitivity at diagnosis, suggesting that survival, proliferative, and immune escape programs are already established. Clinically, these patients frequently present with high tumor burden, bulky disease, elevated LDH, extranodal involvement, and rapidly progressive symptoms [7].

**FIGURE 1:**
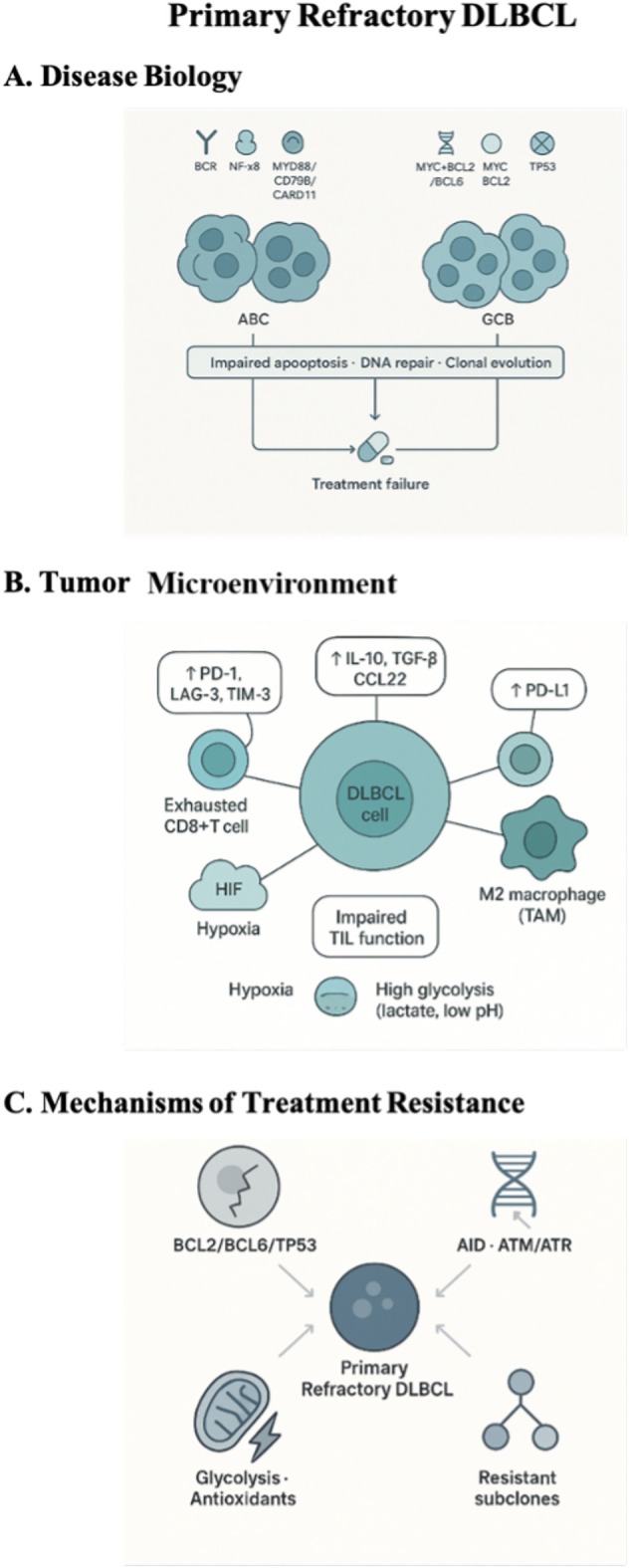
Integrated pathobiology of primary refractory diffuse large B‐cell lymphoma (DLBCL). (A) Intrinsic disease biology in ABC‐ and GCB‐DLBCL, highlighting key signaling alterations (BCR/NF‐κB, MYD88/CD79B/CARD11, double‐hit/double‐expressor, TP53) that converge on impaired apoptosis and DNA repair. (B) Immunosuppressive tumor microenvironment with exhausted T cells, Tregs, M2 macrophages, inhibitory checkpoints, and metabolic stress (hypoxia, high glycolysis) promoting immune escape. (C) Main mechanisms of treatment resistance, including apoptotic dysregulation, enhanced DNA repair, metabolic reprogramming, and clonal evolution leading to a primary refractory phenotype.

An overview of the biological determinants of primary refractoriness—including disease biology, microenvironment interactions, and resistance mechanisms—is shown in Figure 1.

DLBCL comprises two major cell‐of‐origin subtypes with distinct molecular characteristics. The activated B‐cell–like (ABC) subtype is enriched among primary refractory cases and is driven by chronic B‐cell receptor (BCR) signaling [8], constitutive NF‐κB activation, and recurrent mutations in MYD88L265P, CD79B, and CARD11 [9]. These pathways provide potent anti‐apoptotic signals and promote resistance to cytotoxic stress. ABC tumors also depend on IRF4 and JAK/STAT signaling, further contributing to refractoriness. The germinal center B‐cell–like (GCB) subtype includes biologically heterogeneous tumors. Among these, DLBCL with MYC and BCL2 rearrangements, defined by MYC plus BCL2 and/or BCL6 rearrangements, represent the most aggressive forms and respond poorly to standard chemotherapy. DLBCL NOS with immunohistochemical overexpression of MYC and BCL2 without gene rearrangements (the “old” double‐expressor) display intermediate aggressiveness and are also overrepresented in PR‐DLBCL. Across both categories, additional recurrent alterations—particularly TP53 mutations—impair apoptosis, disrupt DNA‐damage responses, and promote clonal adaptation under treatment pressure [10].

High glycolytic activity of lymphoma cells results in lactate accumulation and acidosis, further impairing tumor‐infiltrating lymphocyte function. Hypoxia, common in bulky or rapidly proliferating disease, activates hypoxia‐inducible pathways that upregulate PD‐L1, promote angiogenesis, and enhance metabolic adaptation. These hypoxic niches reshape stromal and immune components toward tumor‐promoting phenotypes. Defective antigen presentation is another hallmark of PR‐DLBCL. Loss of β2‐microglobulin and epigenetic repression of major histocompatibility complex (MHC) class I and II molecules reduce tumor recognition by cytotoxic T lymphocytes. Upregulation of immune‐checkpoint ligands, especially PD‐L1, further exacerbates immune evasion and contributes to resistance to immunotherapeutic strategies [11]. Marked spatial and clonal heterogeneity within the TME allows coexistence of subclones with variable therapeutic sensitivities. Protective niches, rich in stromal and immunosuppressive elements, enable resistant subpopulations to persist during therapy and expand rapidly upon treatment failure. This dynamic interplay between malignant cells and the TME is a critical determinant of primary refractoriness and poor clinical outcome [12].

Early treatment resistance in primary refractory DLBCL arises from a complex interplay of intrinsic molecular abnormalities and adaptive cellular mechanisms. Apoptotic dysregulation is prominent: BCL2 overexpression, BCL6 dysregulation, and TP53 inactivation blunt DNA‐damage–induced apoptosis, enabling survival despite intensive chemotherapy [1]. Enhanced DNA repair capacity also reduces frontline treatment efficacy. Upregulation of activation‐induced cytidine deaminase, activation of ataxia‐telangiectasia mutated (ATM) and ATM and Rad3‐related (ATM/ATR)‐mediated DNA repair pathways, and chromatin remodeling changes accelerate repair of anthracycline‐ and alkylator‐induced DNA damage. Metabolic reprogramming further contributes to chemoresistance. Increased glycolytic flux, altered mitochondrial biogenesis, and elevated antioxidant defenses allow tumor cells to withstand oxidative stress and sustain proliferation despite therapeutic insult [7, 13]. Finally, clonal evolution under therapeutic pressure selects for resistant subclones. Sensitive populations are eliminated, while pre‐existing or therapy‐induced resistant clones expand, leading to rapid progression and treatment failure.

The tumor microenvironment (TME) plays a central role in driving immune dysfunction and sustaining lymphoma cell survival. It is typically immunosuppressive, enriched with exhausted CD8^+^ T cells, regulatory T cells (Tregs), and M2‐polarized tumor‐associated macrophages (TAMs). These populations secrete immunomodulatory cytokines such as IL‐10, TGF‐β, and CCL22, suppressing cytotoxic immunity. Exhausted T cells frequently express inhibitory receptors, including Programmed‐death 1 (PD‐1), Lymphocyte Activation gene 3 (LAG‐3), and T‐cell immunoglobulin and mucin‐domain containing‐3 (TIM), leading to impaired effector function and defective immunologic synapse formation [12].

## 3 Defining “Unsuitable” for CAR T‐Cell Therapy

**FIGURE 2:**
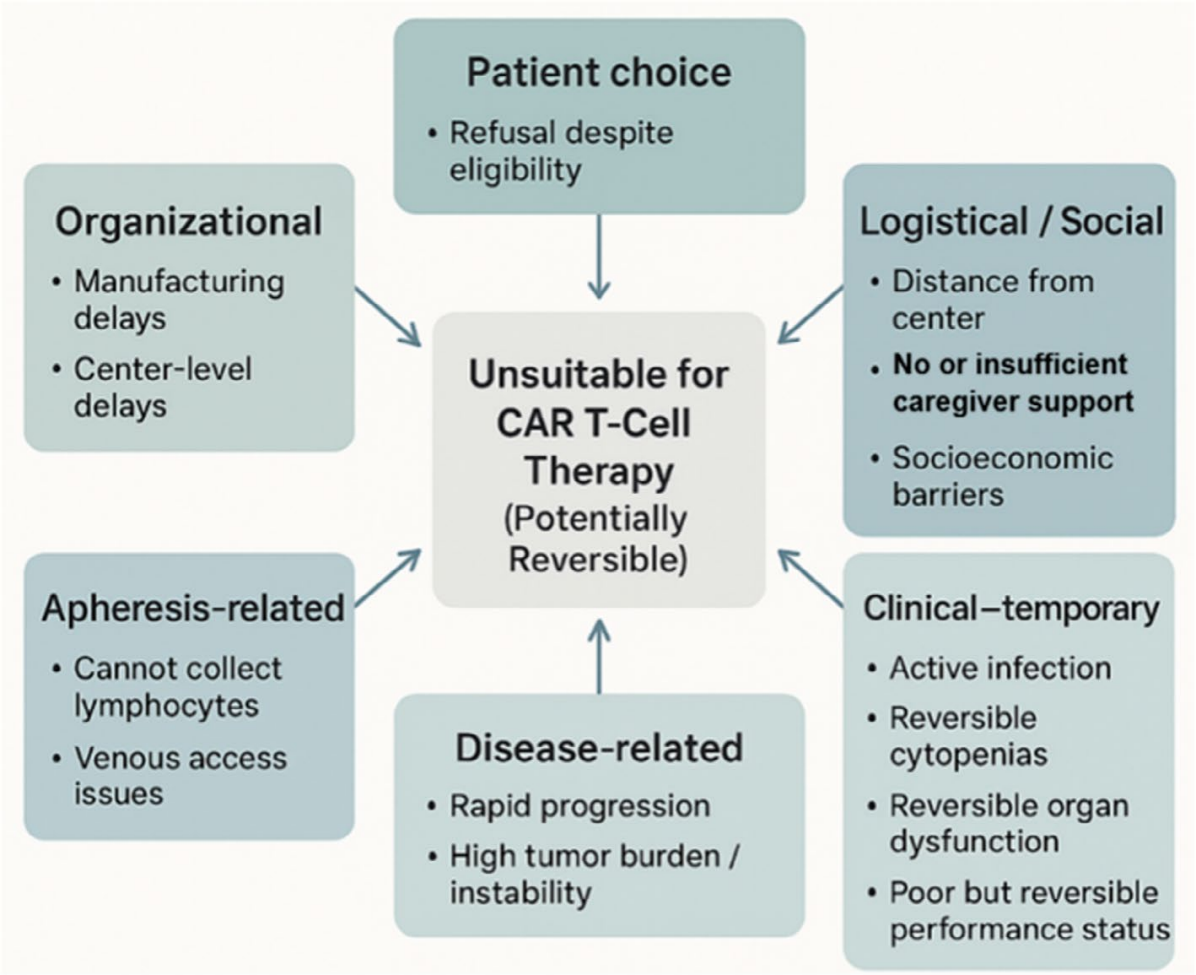
Conditions of unsuitability for CAR T‐cell therapy. Conceptual framework illustrating how potentially reversible factors can render an otherwise eligible patient unsuitable for CAR T‐cell therapy. The figure groups these factors into patient‐related, logistical/social, clinical, disease‐related, apheresis‐related, and organizational domains, emphasizing the multidimensional and often overlapping nature of barriers that must be systematically identified and, where possible, corrected in patients with primary refractory DLBCL.

The concept of “unsuitability” for CAR T‐cell therapy encompasses a diverse group of patients who, despite meeting disease‐related eligibility criteria, cannot proceed with treatment because of potentially reversible clinical, logistical, or organizational limitations. Distinguishing these individuals from those who are fully eligible or permanently ineligible is essential, as many of these barriers can be overcome with appropriate medical intervention, stabilization, or system‐level adjustments. Recognition of the specific domain of unsuitability—patient choice, logistical/social, clinical, disease‐related, apheresis‐related, or organizational (Figure 2)—is crucial to tailoring both supportive measures and alternative treatment strategies.

### 3.1 Distinguishing Eligible vs. Unsuitable vs. Ineligible

Clear differentiation between eligible, unsuitable, and ineligible patients is critical for accurate clinical decision‐making in the context of CAR T‐cell therapy for primary refractory DLBCL. Eligible patients are those who meet disease‐ and treatment‐related criteria for CAR T‐cell therapy and can safely undergo leukapheresis, manufacturing, and infusion, with adequate organ function, performance status, and logistical support. Importantly, the assessment of eligibility and suitability should be performed by a specialized CAR T‐cell team at an accredited CAR T center, rather than by community clinicians alone. The designation of a patient as *unsuitable* for CAR T‐cell therapy should therefore reflect a multidisciplinary evaluation by CAR T physicians, taking into account both clinical and practical factors that may preclude immediate treatment. This distinction is essential to avoid inappropriate exclusion or delayed referral and to ensure that patients who are temporarily unsuitable are promptly re‐evaluated when barriers are resolved. These individuals may require bridging therapy but have no prohibitive barriers to proceeding with CAR T‐cell treatment [14, 15].

By contrast, unsuitable patients are those who satisfy disease‐related eligibility criteria but cannot undergo CAR T‐cell therapy at the time of evaluation owing to potentially reversible clinical, logistical, or organizational factors. This category includes patients with rapidly progressive disease requiring immediate cytoreductive therapy, transient organ dysfunction, uncontrolled infection, severe but reversible cytopenias, or a temporary deterioration in performance status that is expected to improve with disease control. Unsuitability may also arise from external circumstances such as limited caregiver support, inability to remain near a CAR‐T center, socioeconomic constraints, patient refusal, or delays related to manufacturing capacity or institutional resources. These barriers do not permanently preclude CAR T‐cell therapy but necessitate alternative therapeutic strategies to stabilize disease or address modifiable limitations.

Within the group of patients classified as unsuitable for CAR T‐cell therapy, a further distinction should be made between those who are temporarily unsuitable but potentially convertible to CAR T and those who are functionally unsuitable because of rapidly progressive disease biology. The former category includes patients in whom unsuitability is driven by reversible clinical factors—such as transient organ dysfunction, active infection, severe cytopenias, or poor performance status related to tumor burden—and who may regain eligibility following effective disease control or supportive interventions. In contrast, a subset of patients presents with extremely aggressive disease kinetics characterized by explosive tumor growth, early chemoresistance, and rapid clinical deterioration, making CAR T‐cell therapy unfeasible in practice despite theoretical reversibility of clinical parameters. In these cases, the time of disease progression may exceed the time required for leukapheresis, manufacturing, and infusion, making immediate disease control a clinical priority. In patients with rapidly progressive disease, the therapeutic goal initially shifts toward urgent cytoreduction and short‐term disease stabilization. However, this strategy does not necessarily preclude subsequent access to CAR T‐cell therapy. Indeed, the use of novel agents and combination approaches (e.g., bispecific antibodies and antibody–drug conjugates) may allow effective disease control and, in selected cases, enable later conversion to cellular therapy.

In contrast, ineligible patients have permanent or non‐reversible contraindications that preclude CAR T‐cell therapy irrespective of disease status or supportive measures. These may include irreversible organ failure, uncontrolled comorbidities, prohibitive frailty, or active secondary malignancies. For these patients, CAR T‐cell therapy is not a feasible option, and long‐term management must rely on alternative therapeutic modalities [14].

Eligibility for CAR T‐cell therapy should be restricted to patients with truly permanent or non‐reversible contraindications. Objective criteria may include a persistently poor performance status (ECOG ≥ 3) not driven by tumor burden and severe, irreversible organ dysfunction. However, it should be acknowledged that selected patients with chronic renal failure requiring dialysis, decompensated liver disease, or severe cardiac impairment (left ventricular ejection fraction < 30%–35%) have been successfully treated with CAR T‐cell therapy in specific clinical contexts, as reported in the literature. Therefore, these conditions should not be considered absolute contraindications but rather factors requiring individualized multidisciplinary assessment. Additional criteria for ineligibility include uncontrolled comorbidities with limited life expectancy, active secondary malignancies requiring systemic treatment, or profound frailty or cognitive impairment precluding safe post‐infusion monitoring. By contrast, cytopenias, transient organ dysfunction, or performance status decline attributable to lymphoma should be regarded as indicators of temporary unsuitability rather than definitive ineligibility. This distinction is critical to avoid excluding patients who may regain eligibility after disease control or supportive intervention [16].

### 3.2 Causes of Unsuitability

Patients deemed unsuitable for CAR T‐cell therapy represent a heterogeneous group in whom barriers to treatment are potentially reversible but clinically significant at the time of evaluation. These barriers can be broadly categorized into clinical, logistical/social, patient‐driven, and organizational factors. Figure 2 summarizes the main domains of potentially reversible unsuitability for CAR T‐cell therapy—ranging from patient choice and logistical or social barriers to temporary clinical, disease‐related, apheresis‐related, and organizational factors—that should be systematically assessed in patients with primary refractory DLBCL.

#### 3.2.1 Clinical Reasons

Clinical causes of unsuitability for CAR T‐cell therapy often arise from acute or unstable medical conditions that temporarily compromise the safety or feasibility of treatment. A frequent situation is a rapidly progressive disease, in which the clinical deterioration occurs so swiftly that there is insufficient time to complete leukapheresis and CAR‐T manufacturing, although, many CAR‐T products have a shorter door‐to‐door time nowadays, like 15–20 days; in such cases, immediate cytoreductive therapy becomes unavoidable. Active infections also represent a major temporary barrier: bacterial, fungal, or viral illnesses must be adequately controlled before the patient can safely undergo immunosuppressive procedures such as lymphodepletion and CAR T‐cell infusion. Reversible organ dysfunction constitutes another important cause of temporary unsuitability: acute kidney injury, hepatic impairment in the absence of chronic liver disease, or episodes of cardiac decompensation may render treatment unsafe but can improve with appropriate medical interventions [17]. Similarly, severe yet reversible cytopenias, frequently related to prior myelotoxic therapy, may preclude safe leukapheresis or increase the risk of complications during conditioning therapy. Finally, some patients present with a markedly reduced performance status, typically ECOG ≥ 3, reflecting the debilitating impact of uncontrolled lymphoma rather than fixed comorbidities; these patients may regain eligibility once disease control is achieved [18].

#### 3.2.2 Logistical and Social Barriers

Logistical and social factors are major determinants of temporary unsuitability for CAR T‐cell therapy and often intersect with issues of healthcare access and structural inequity. Because CAR T‐cell therapy is delivered only in specialized, accredited centers, patients who live at considerable distances from such facilities may face substantial challenges in reaching and remaining near the treating institution for the prolonged monitoring period required after infusion. This geographic barrier is particularly pronounced for individuals with limited mobility, inadequate transportation resources, or dependence on social services [19]. Equally critical is the requirement for a reliable caregiver, whose presence is essential for post‐infusion surveillance, early recognition of toxicities such as cytokine release syndrome or neurotoxicity, and adherence to follow‐up visits. Patients lacking consistent caregiver support, whether due to social isolation, family constraints, or socioeconomic vulnerability, are often deemed unsuitable until appropriate support systems are established [20]. Additionally, the financial burden associated with travel, temporary housing, work absence, and ancillary costs may exceed a patient's economic capacity, further restricting access despite clinical eligibility.

#### 3.2.3 Patient‐Driven Factors

Patient‐driven factors also contribute meaningfully to unsuitability for CAR T‐cell therapy. Some patients refuse treatment because of apprehensions about potential toxicities, hospitalization requirements, or the unpredictable clinical trajectory associated with cellular immunotherapies. Others may decline based on personal values, prior negative experiences with intensive treatments, or concerns about quality of life during and after therapy. In certain cases, misunderstanding of the therapeutic process or unrealistic expectations may shape decision‐making, underscoring the need for robust patient education and shared decision‐making. Other reasons could be the burden on family or caregivers and apprehension about prolonged time off work. Although refusal may be reversible if concerns are adequately addressed, it nonetheless renders the patient temporarily unsuitable at the time of evaluation [21, 22].

#### 3.2.4 Impact of Unsuitability on Prognosis and Treatment Planning

Temporary unsuitability for CAR T‐cell therapy has important implications for both prognosis and therapeutic decision‐making in primary refractory DLBCL. Patients who cannot proceed directly to CAR‐T frequently require bridging or alternative therapy to control disease while efforts are made to overcome the identified barriers [23]. However, the efficacy of available salvage options in this setting is often limited, and the risk of further clinical deterioration during the waiting period is substantial. Delays in accessing CAR T‐cell therapy may permit continued tumor evolution and selection of more resistant subclones, thereby reducing the likelihood of eventual treatment success [4, 24]. Moreover, the need to prioritize short‐term disease control may preclude the use of optimal long‐term strategies, compelling clinicians to adopt regimens that stabilize rather than fundamentally alter the disease trajectory [24]. Psychosocial stress, financial strain, and cumulative toxicity from interim therapies further compound clinical complexity. As a result, unsuitability not only affects the immediate therapeutic pathway but may also compromise the probability of achieving durable remission.

## 4 Therapeutic Options for Patients Unsuitable for CAR T‐Cell Therapy

**FIGURE 3:**
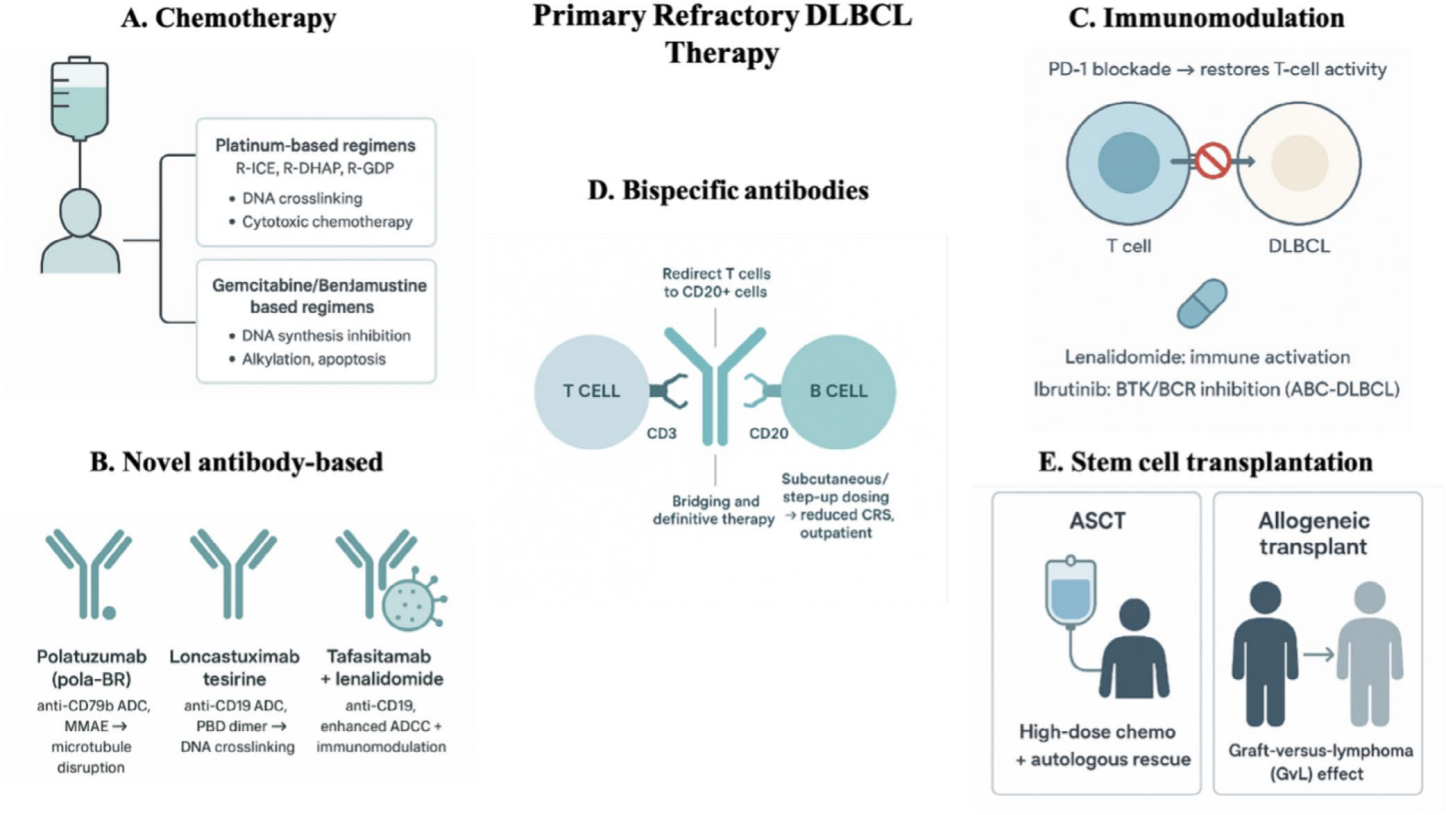
Therapeutic options for CAR T‐unsuitable primary refractory DLBCL. (A) Conventional salvage chemotherapy: Platinum‐based regimens (R‐ICE, R‐DHAP, R‐GDP) inducing DNA crosslinking and cytotoxicity, and gemcitabine/bendamustine‐based regimens acting via DNA synthesis inhibition and alkylation‐induced apoptosis. (B) Novel antibody‐based therapies: Polatuzumab vedotin–based regimens (anti‐CD79 b ADC delivering MMAE for microtubule disruption), loncastuximab tesirine (anti‐CD19 ADC with PBD dimer–mediated DNA crosslinking), and tafasitamab plus lenalidomide (anti‐CD19 with enhanced ADCC and immunomodulation). (C) Checkpoint inhibitors and immunomodulation: PD‐1 blockade restores T‐cell antitumor activity, and lenalidomide plus ibrutinib in ABC‐DLBCL provides immune activation and BTK/BCR pathway inhibition. (D) CD20 × CD3 bispecific antibodies: T‐cell redirection toward CD20^+^ lymphoma cells, with subcutaneous or step‐up dosing reducing CRS risk and enabling outpatient use as bridging or definitive therapy. (E) Stem cell transplantation: Autologous stem cell transplantation (ASCT) using high‐dose chemotherapy with autologous rescue and allogeneic transplantation leveraging graft‐versus‐lymphoma effects from donor immune cells.

Patients with PR‐DLBCL who are unsuitable for CAR T‐cell therapy constitute a highly vulnerable population requiring timely, effective, and biologically rational interventions (Table 1, Figure 3). Given the narrow window for disease control, therapeutic strategies must balance rapid cytoreduction with toxicity preservation of organ function and, when feasible, restoration of eligibility for advanced therapies. Conventional salvage chemotherapy remains relevant, particularly when immediate disease debulking is required or when clinical instability precludes immunotherapeutic approaches [30]. Although associated with limited long‐term efficacy in primary refractory disease, these regimens continue to play a critical role in short‐term disease control and symptom palliation.

**TABLE 1:** Comparative efficacy and safety profile of therapeutic strategies for primary refractory DLBCL unsuitable for CAR T‐cell therapy.

Strategy	Therapies	Efficacy outcomes	Toxicity profile	References
Conventional salvage chemotherapy	Platinum‐based; Gemcitabin/bendamustine‐based	ORR 25%–40%; CR 10%–15%; median PFS 2–4 months; median OS < 6–8 months	Hematologic AEs; renal toxicity; neuropathy.	[4, 5]
Antibody–drug conjugates and antibody‐based combinations	Polatuzumab vedotin + BR; Loncastuximab tesirine; Tafasitamab + lenalidomide	Pola‐BR: ORR 45%–60%, CR 35%–40%, median PFS 6–9 months, OS ~10–12 months; Loncastuximab: ORR 45%–50%, CR 20%–25%, PFS 4–6 months; Tafasitamab‐lenalidomide: ORR 55%–60%, CR 40%–45%, median PFS ~12 months, OS > 30 months	Neutropenia; infections; peripheral neuropathy (polatuzumab) mostly grade 1–2; edema/effusions and liver enzyme elevations with loncastuximab.	[25]
Checkpoint inhibitors and immunomodulatory strategies	PD‐1 inhibitors; Lenalidomide ± rituximab; Ibrutinib‐based combinations	PD‐1 inhibitors: ORR < 10%–15%, CR < 5%, PFS 2–3 months; Lenalidomide‐based: ORR 30%–40%, CR 15%–25%, PFS 4–6 months; Ibrutinib‐based: ORR 35%–45%	Generally favorable safety; immune‐related AEs; lenalidomide‐associated cytopenias 25%–40%, rash/fatigue; ibrutinib‐related atrial fibrillation 5%–10%, bleeding 10%–20%	[26, 27]
CD20 × CD3 bispecific antibodies	Epcoritamab; Glofitamab; Mosunetuzumab	ORR 55%–70%; CR 35%–45%; median PFS 6–10 months; median OS often > 12 months	CRS; ICANS; neutropenia; infections	[28]
Stem cell transplantation	Autologous SCT; Allogeneic SCT	ASCT: durable remission in ~30%–40% of chemosensitive patients; 2‐year PFS < 20% in refractory disease.	ASCT: grade ≥ 3 mucositis, infections, cytopenias > 70%–80%.	[29]
		Allo‐SCT: 2‐year OS ~30%–40%	Allo‐SCT: acute GVHD 30%–50%, chronic GVHD 40%–60%.	

### 4.1 Conventional Salvage Strategies

Conventional salvage chemotherapy has long been the cornerstone of treatment for PR‐DLBCL and is frequently used in patients unsuitable for CAR T‐cell therapy as a bridging strategy to stabilize rapidly progressive disease, improve performance status, or reverse temporary clinical contraindications. Despite modest activity in primary refractoriness, these regimens often represent the only immediately available option capable of providing rapid cytoreduction. Regimen selection—platinum‐based versus gemcitabine‐ or bendamustine‐containing combinations or therapies—is guided by prior treatment exposures, organ function, comorbidities, and therapeutic goals, either as a definitive strategy or to restore eligibility for advanced therapies [31]. The main conventional salvage approaches are summarized in Figure 3A.

Platinum‐containing regimens, including R‐ICE (rituximab plus ifosfamide, carboplatin, and etoposide), R‐DHAP (rituximab plus dexamethasone, high‐dose cytarabine, and cisplatin), R‐GemOx (rituximab plus gemcitabine and oxaliplatin), and R‐GDP (rituximab plus gemcitabine, dexamethasone, and cisplatin) induce cytotoxicity through DNA crosslinking and nucleic acid disruption and are preferred when urgent disease control is required, such as in bulky or symptomatic disease [32].

In PR‐DLBCL, however, outcomes remain poor, with overall response rates (ORR) of 25%–40%, complete response (CR) rates of 10%–15%, median progression‐free survival (PFS) of 2–4 months, and median overall survival (OS) below 6–8 months without transplant consolidation [33].

Grade ≥ 3 hematologic occurs in 60%–80% of patients, with febrile neutropenia in 10%–25%; non‐hematologic toxicities include renal impairment (10%–20%), neuropathy (10%–15%), and gastrointestinal adverse events (20%–30%), while treatment‐related mortality is reported in 2%–5% [34].

Regimens such as R‐GemOx, GemOx, or BR offer more favorable tolerability, particularly in older or frail patients, while maintaining cytoreductive activity [35]. Although efficacy remains limited (ORR 20%–35%, CR 10%–20%), these approaches can improve symptoms, enhance performance status, and facilitate transition to subsequent therapies, including bispecific antibodies [31].

### 4.2 Novel Antibody‐Based and Other Therapies

Novel antibody‐based therapies have broadened treatment options for patients with PR‐DLBCL who are unsuitable for CAR T‐cell therapy. By targeting key surface antigens and exploiting antibody‐drug conjugation, immune‐cell recruitment, and immunomodulation, these agents provide clinically meaningful benefit in a population with limited alternatives [25]. Although their efficacy is generally inferior to CAR T‐cell therapy, their rapid availability, outpatient feasibility, and manageable toxicity profiles make them integral to current treatment algorithms (Figure 3B).

Polatuzumab vedotin is an antibody–drug conjugate (ADC) targeting CD79b, a B‐cell receptor component highly expressed in DLBCL. After internalization, it releases monomethyl auristatin E, disrupting microtubules and inducing apoptosis independently of p53, a feature relevant to high‐risk subtypes such as double‐expressor and double‐hit lymphomas [36]. The combination of polatuzumab, bendamustine, and rituximab (pola‐BR) has demonstrated superior efficacy over bendamustine‐rituximab alone, which is advantageous in patients with high tumor burden or symptomatic disease. Real‐world data confirm activity in frail, elderly, or comorbid patients often excluded from trials, supporting its use in the CAR‐T‐unsuitable setting. Reported outcomes include an ORR of 45%–60%, CR rates of 35%–40%, median PFS of 6–9 months, and a median OS of 10–12 months [37]. Toxicity is mainly hematologic, with grade ≥ 3 neutropenia in 40%–50%, thrombocytopenia in 30%–40%, infections in 15%–25%, and mostly low‐grade peripheral neuropathy; severe neuropathy is uncommon.

Loncastuximab tesirine is a next‐generation ADC targeting CD19 and conjugated to a pyrrolobenzodiazepine dimer that induces DNA crosslinking. This mechanism enables activity in tumors with multidrug resistance phenotypes, defective apoptosis, or high proliferation, features common in PR‐DLBCL [38]. In the LOTIS‐2 study, loncastuximab achieved ORRs of about 45%–50%, with CR rates around 20%–25% in heavily pretreated patients, including those with PR‐DLBCL or prior CAR T‐cell exposure. Median duration of response is ~10 months, with median PFS of 4–6 months and OS of 9–10 months [39].

Grade ≥ 3 cytopenias occur in 25%–35%, while characteristic non‐hematologic toxicities include edema or effusions (15%–25%), liver enzyme elevations (10%–20%), and cutaneous or photosensitivity reactions (10%–15%); discontinuation rates remain below 10%–15%.

The combination of tafasitamab and lenalidomide represents a biologically synergistic, immune‐based strategy. Tafasitamab is an Fc‐engineered anti‐CD19 antibody that enhances natural killer (NK) cell–mediated antibody‐dependent cellular cytotoxicity (ADCC) and macrophage‐mediated phagocytosis, with lenalidomide, which enhances T‐ and NK‐cell function and favorably modulates the tumor microenvironment [40]. In the phase II L‐MIND study, this regimen produced durable responses, particularly in non–double‐hit DLBCL and patients with lower tumor burden and indolent disease kinetics. Transition to tafasitamab monotherapy after the combination phase allows sustained therapy with favorable tolerability [41]. This approach is well suited for patients unable to tolerate intensive chemotherapy or frequent hospital visits.

Reported outcomes include ORRs of 55%–60%, CR rates of 40%–45%, median PFS of ~12 months, and median OS exceeding 30 months in responders.

Toxicities are generally manageable, with grade ≥ 3 neutropenia in 40%–50%, thrombocytopenia or anemia in 20%–30%, and mainly low‐grade fatigue (30%–40%), diarrhea (15%–25%), and rash (10%–20%); severe infections occur in ~10%–15%.

### 4.3 Checkpoint Inhibitors, Immunomodulation and BTK Inhibitors

Immune checkpoint inhibitors have shown limited activity in unselected DLBCL, largely due to the immune‐cold tumor biology and frequent defects in antigen presentation. Nevertheless, PD‐1 blockade and immunomodulatory approaches remain relevant for CAR T‐ineligible patients when applied to biologically defined subgroups or within combination strategies designed to restore immune competence(Figure 3C) [42].

Clinical benefit from PD‐1 inhibitors appears confined to primary mediastinal B‐cell lymphoma (PMBCL) and tumors with high PD‐L1/PD‐L2 expression or 9p24.1 alterations [26].

Outside these niches, efficacy is modest, with ORR generally < 10%–15%, CR < 5%, and median PFS of 2–3 months. Toxicity is usually manageable, with immune‐related adverse events in 15%–30% of patients and grade ≥ 3 events in 5%–10% [43].

Lenalidomide exerts pleiotropic immunomodulatory and antitumor effects in ABC‐DLBCL through IRF4 degradation, NF‐κB inhibition, and enhancement of T‐cell and NK‐cell activity.

In the phase 3 ECHELON‐3 trial, BV + lenalidomide + rituximab was compared with placebo + lenalidomide + rituximab in patients with R/R DLBCL. The combination significantly improved overall survival (13.8 vs. 8.5 months) and progression‐free survival (4.2 vs. 2.6 months). Objective response rate was higher with BV‐based therapy (64% vs. 42%), with increased complete responses (40% vs. 19%) [27].

Ibrutinib targets BCR signaling and shows preferential activity in molecularly defined ABC‐DLBCL, particularly in tumors harboring MYD88 L265P and CD79B mutations. While single‐agent activity is limited, combination regimens incorporating ibrutinib have demonstrated clinically meaningful responses [44]. Toxicities include atrial fibrillation (5%–10%), bleeding (10%–20%), infections (15%–25%), and hypertension, particularly in older or frail patients.

### 4.4 Bispecific Antibodies

CD20 × CD3 bispecific antibodies represent one of the most impactful therapeutic innovations for PR‐DLBCL, particularly in patients unsuitable for CAR T‐cell therapy. By simultaneously engaging CD3 on T cells and CD20 on malignant B cells, these agents induce rapid, MHC‐independent cytotoxicity and overcome multiple immune evasion mechanisms. However, their activity is strictly dependent on the presence of CD20 expression on tumor cells. It should be noted that loss or downregulation of CD20 may occur after prior anti‐CD20–based therapies, with reported rates ranging from approximately 10%–20% of relapsed/refractory cases [28, 45]. Their predictable safety profile, outpatient administration, and immediate availability make them well‐suited for patients with comorbidities, logistical constraints, or transient clinical instability. As illustrated in Figure 3D, CD20 × CD3 bispecific antibodies can be used as bridging or definitive therapy. Across studies, ORR ranged from 55% to 70%, with CR rates of 35%–45%, median PFS of 6–10 months, and median OS frequently exceeded 12 months.

Cytokine release syndrome (CRS) is the most characteristic adverse event, occurring in 50%–70% of patients, but grade ≥ 3 events are uncommon (5%–10%) due to step‐up dosing and premedication; CRS is typically early, low‐grade, and self‐limited.

Immune effector cell–associated neurotoxicity syndrome (ICANS) is rare (< 5% of patients, usually grade 1–2). Other toxicities include cytopenias (grade ≥ 3 neutropenia in 20%–40%), infections (15%–30%), and injection‐site reactions with subcutaneous formulations [45, 46].

An additional consideration is the potential impact of prior anti‐CD20 exposure on the efficacy of CD20 × CD3 bispecific antibodies. Prolonged or repeated treatment with anti‐CD20 monoclonal antibodies may lead to partial CD20 downregulation or antigen masking, which could reduce target density and impair T‐cell–mediated cytotoxicity. This issue is particularly relevant in heavily pretreated and primary refractory disease, where selective pressure from prior therapies may favor CD20‐low or CD20‐negative subclones. Although bispecific antibodies can retain activity in this setting, assessment of CD20 expression and prior treatment history may be important to optimize patient selection and sequencing strategies.

Epcoritamab is a subcutaneously administered CD20 × CD3 bispecific antibody combining high efficacy with favorable tolerability and outpatient administration. In relapsed/refractory DLBCL—including post‐CAR T‐cell therapy, epcoritamab achieves ORR > 60%, with CR rates of ~40%, many of which are durable [28]. Its ease of administration and rapid cytoreduction make it a practical option for CAR T–ineligible patients. Early frontline combinations with R‐CHOP, Pola‐CHP, or DA‐R‐EPOCH have yielded very high CR rates, suggesting potential expansion into earlier treatment lines.

Glofitamab is a 2:1 CD20 × CD3 bispecific antibody with enhanced avidity and cytotoxic potency. Administered intravenously with an obinutuzumab pretreatment dose to mitigate CRS, glofitamab has demonstrated CR rates exceeding 35%–40%, including in heavily pretreated and CAR T–exposed patients [28]. Its fixed‐duration treatment schedule (typically 12 cycles) is advantageous for patients unable or unwilling to pursue prolonged therapy, although inpatient monitoring may be required during initial dosing.

Pre‐treatment with Obinutuzumab does not affect Glofitamab efficacy because Glofitamab is a bispecific antibody that redirects T cells to CD20‐expressing B cells, inducing MHC‐independent cytotoxicity. This mechanism is largely independent of prior CD20‐targeted therapy, and clinical data have shown that responses to Glofitamab are maintained even in patients previously exposed to anti‐CD20 antibodies. Moreover, any transient reduction in CD20 expression after Obinutuzumab does not appear to significantly impair the ability of Glofitamab to engage remaining CD20‐positive tumor cells and mediate effective cytotoxicity.

Importantly, the phase III STAR‐GLO trial specifically addressed the population of patients with relapsed/refractory DLBCL who are ineligible for CAR T‐cell therapy. In this study, the combination of glofitamab with GemOx demonstrated superior efficacy compared with GemOx alone, with significantly higher response rates and improved progression‐free survival, establishing a CD20 × CD3 bispecific antibody–based regimen as a new standard option in this clinically vulnerable population. Since in the STAR‐GLO trial the treatment durations differed between arms (Gem‐Ox for 6 months vs. Glofitamab for 1 year), it could contribute to the observed differences in efficacy. Therefore, while prior Obinutuzumab is unlikely to affect Glofitamab activity, the longer treatment exposure may also play a role in the reported outcomes. However, these data provide prospective, randomized evidence supporting the use of bispecific antibodies not only as bridging strategies but also as definitive therapy for patients who cannot receive CAR T‐cell therapy. The availability of an off‐the‐shelf, T‐cell–engaging immunotherapy with a rapid onset of action and a manageable toxicity profile directly addresses the key limitations faced by CAR‐T, including time constraints, comorbidities, and logistical barriers. Therefore, CD20 × CD3 bispecific antibodies should be considered a preferred immunotherapeutic option for patients who are unsuitable or ineligible for CAR T‐cell therapy, particularly when rapid cytoreduction is required, and long‐term disease control remains a realistic goal [46].

Mosunetuzumab, the first approved CD20 × CD3 bispecific antibody, has a favorable CRS profile allowing largely outpatient administration. While approved for follicular lymphoma, mosunetuzumab has demonstrated meaningful activity in DLBCL, particularly when combined with CHOP or Pola‐CHP, with early‐phase studies reporting high response rates and encouraging durability, supporting potential future frontline applications in high‐risk patients [28].

### 4.5 Stem Cell Transplantation

Stem cell transplantation remains an option for selected patients with PR‐DLBCL who are unsuitable for CAR T‐cell therapy, particularly when chemosensitivity can be achieved [47]. The principles of autologous and allogeneic transplantation in this setting are summarized in Figure 3E.

Autologous stem cell transplantation (ASCT), once the standard of care for chemosensitive relapsed DLBCL, now plays a more limited role but remains relevant for patients who are temporarily rather than permanently ineligible for CAR T‐cell therapy. Outcomes depend critically on chemosensitivity: patients attaining at least a partial response to platinum‐ or gemcitabine‐based regimens may achieve durable remissions in approximately 30%–40% of cases, whereas truly refractory disease is associated with poor outcomes, with 2‐year PFS rates below 20%. ASCT may be particularly relevant in healthcare systems with limited CAR‐T access or prolonged manufacturing timelines [29]. Toxicity remains substantial, with grade ≥ 3 mucositis, infections, and cytopenias occurring in over 70%–80% of patients and transplant‐related mortality of 1%–3%.

Allo‐SCT offers the potential for long‐term disease control through graft‐versus‐lymphoma (GvL) activity and therefore represents a largely theoretical option for patients who relapse after or cannot receive CAR T‐cell therapy. In real‐world practice, there are virtually no patients who are eligible for allo‐SCT but ineligible for CAR T‐cell therapy, given the substantially higher toxicity and fitness requirements associated with transplantation. Although allo‐SCT may induce durable remissions via GvL effects, with a reported 2‐year overall survival of approximately 30%–40%, its role in primary refractory DLBCL remains marginal and confined to highly selected cases. Consequently, allo‐SCT is reserved for highly selected, fit patients with chemosensitive disease who lack access to CAR T‐cell therapy or who relapse after CAR T‐cell treatment [48]. Despite broader eligibility with reduced‐intensity conditioning, allo‐SCT is generally considered only after exhaustion of other immunotherapeutic options or in the presence of adverse molecular features predicting poor response to non‐cellular therapies.

## 5 Future Directions

The therapeutic landscape for DLBCL is undergoing rapid transformation, driven by advances in cellular engineering, immune engagement, and targeted drug development. For patients unsuitable for CAR T‐cell therapy—a historically underserved population—emerging technologies offer the prospect of expanding access, reducing toxicity, and achieving deeper, more durable remissions. Future paradigms will likely combine next‐generation cellular therapies, novel bispecific constructs, and rational small‐molecule combinations, supported by increasingly refined molecular profiling to tailor therapy to tumor biology [28].

**FIGURE 4:**
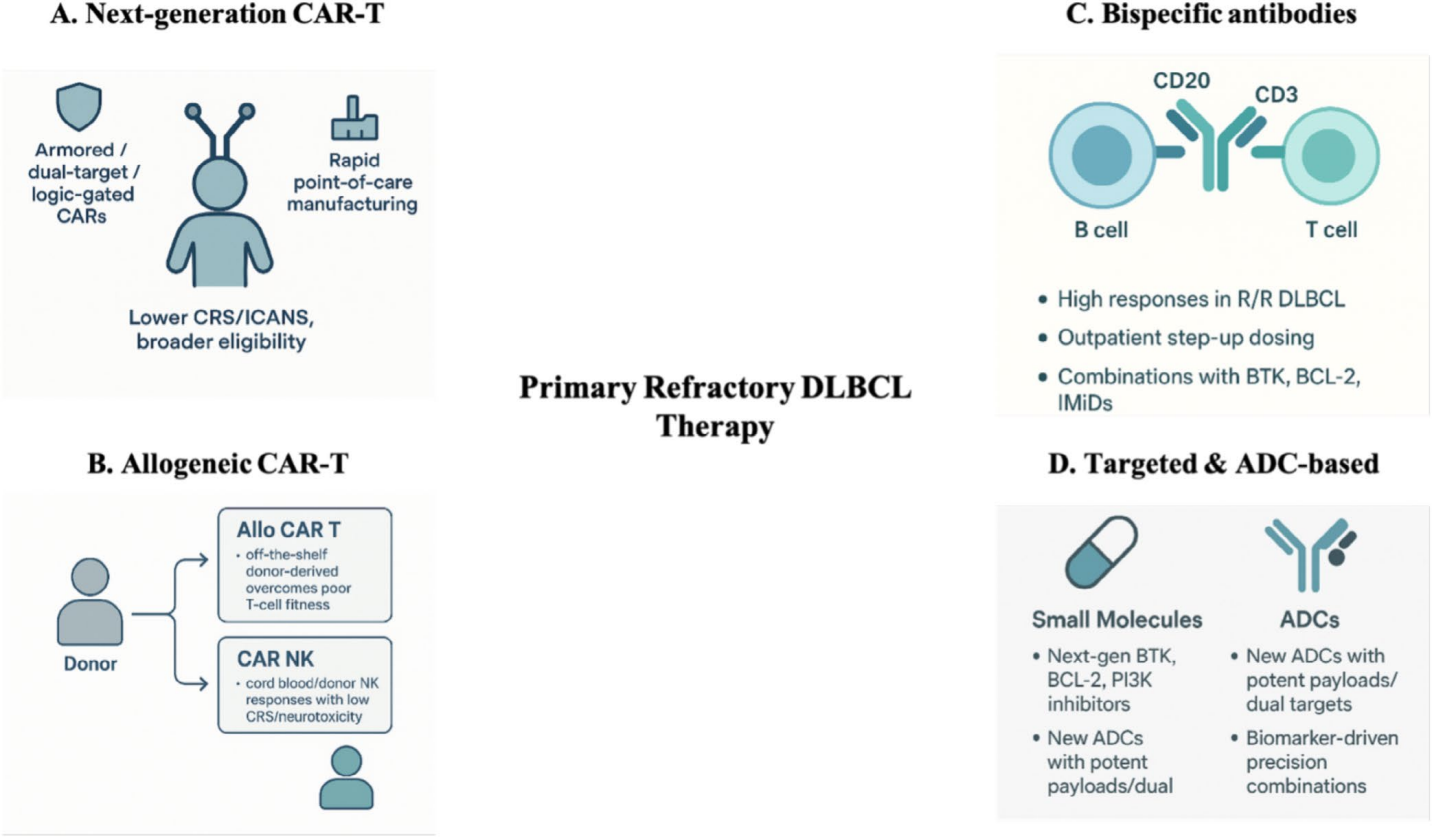
Future therapeutic directions for CAR T‐unsuitable DLBCL. Conceptual overview of emerging strategies for patients unsuitable for CAR T‐cell therapy, including: (A) next‐generation CAR T‐cell constructs (armored, dual‐target, logic‐gated designs with rapid point‐of‐care manufacturing and reduced CRS/ICANS risk); (B) allogeneic cellular therapies, comprising donor‐derived CAR T cells and CAR NK cells as off‐the‐shelf products that bypass poor autologous T‐cell fitness and can deliver responses with low rates of severe immune toxicities; (C) CD20 × CD3 bispecific antibodies providing potent T‐cell redirection with outpatient step‐up dosing and expanding use in rational combinations with BTK, BCL‐2, and immunomodulatory agents; and (D) molecularly targeted agents and novel antibody–drug conjugates, including next‐generation BTK, BCL‐2, and PI3K inhibitors and advanced ADCs, forming the basis of biomarker‐driven precision combinations. All of these approaches aim to achieve deeper, durable remissions and to broaden effective options for patients with PR‐DLBCL who remain unsuitable for CAR T‐cell therapy.

Figure 4 schematically summarizes the main future therapeutic directions for CAR T‐unsuitable DLBCL, including next‐generation CAR T platforms, allogeneic cellular therapies, CD20 × CD3 bispecific antibodies, and emerging targeted and ADC‐based strategies.

Investigation strategies include several next‐generation CAR T‐cell designs aimed at overcoming current limitations in efficacy, safety, and durability. “Armoured” CARs are engineered to secrete immunostimulatory cytokines (e.g., IL‐12, IL‐15) or to express dominant‐negative or switch receptors that modulate inhibitory checkpoint signals, thereby reshaping the immunosuppressive tumor microenvironment. Dual‐target or multiplexed CARs are designed to simultaneously recognize two or more antigens, reducing the risk of antigen‐loss relapse and enhancing tumor clearance. Logic‐gated or “modular” CARs incorporate AND/OR/NOT circuits or inducible domains to improve tumor specificity, minimize off‐tumor toxicity, and allow conditional activation or deactivation of CAR T cells in vivo.

In parallel, innovations in manufacturing—including rapid point‐of‐care or automated platforms—are shortening the interval from leukapheresis to infusion from several weeks to just a few days. This is particularly critical for patients with aggressive or rapidly progressive disease, who might otherwise deteriorate or become temporarily unsuitable while awaiting product release. These advances collectively aim to broaden the applicability, safety, and real‐world feasibility of CAR T‐cell therapy in high‐risk populations.

Refinement of signaling domains and incorporation of intrinsic safety switches are expected to mitigate cytokine release syndrome and neurotoxicity, broadening eligibility to older or comorbid patients and potentially enabling earlier‐line use [49].

Allogeneic cellular therapies represent a complementary paradigm shift for patients who cannot undergo autologous CAR T‐cell therapy because of time constraints, manufacturing failures, or inadequate T‐cell fitness. Off‐the‐shelf allogeneic CAR T and CAR NK products circumvent the need for patient‐derived starting material, often compromised by prior therapy or disease‐related immunosuppression. Systematic reviews of early‐phase trials in relapsed/refractory large B‐cell lymphoma suggest that allogeneic CD19‐directed cellular therapies can achieve substantial response rates with manageable safety profiles, though persistence, immune rejection, and durability of remission remain active areas of investigation.

Allogeneic CAR NK cells offer an additional layer of flexibility and safety. Their innate antitumor activity, lower risk of severe cytokine release or neurotoxicity, and ease of production make CAR NK products a compelling option for frail or clinically unstable patients. In a seminal phase 1/2 trial of HLA‐mismatched cord‐blood–derived anti‐CD19 CAR NK cells (NCT03056339), 8 of 11 patients (73%) with relapsed/refractory CD19^+^ malignancies—4 of whom had lymphoma—responded, including 7 complete remissions, without development of cytokine release syndrome, neurotoxicity, or graft‐versus‐host disease [48, 49]. Subsequent studies confirm that CAR NK platforms are particularly attractive for frail or clinically unstable patients [50].

Bispecific CD20 × CD3 antibodies have already reshaped the treatment continuum for relapsed or refractory DLBCL, and are likely to remain central in future strategies, especially for CAR‐T–unsuitable patients. Next‐generation CD20 × CD3 bispecifics such as glofitamab and epcoritamab have achieved high response rates with largely outpatient step‐up regimens [51]. The field is now moving beyond monotherapy toward rational combinations that integrate bispecific antibodies with BTK inhibitors, BCL‐2 inhibitors, or immunomodulatory agents, deepen responses, and potentially overcome resistance to chemotherapy. For patients unsuitable for CAR T‐cell therapy, such regimens may provide the benefits of T‐cell engagement without the logistical and toxicity burdens associated with individualized cellular manufacturing and prolonged inpatient monitoring.

In parallel, a broad pipeline of molecularly targeted therapies reflects the biological heterogeneity of DLBCL. Refined BTK inhibitors, apoptotic priming strategies with BCL‐2 inhibitors, redesigned PI3K inhibitors, and next‐generation ADCs with optimized payloads and linker technologies are advancing through clinical development [52]. Ultimately, the most promising direction lies in combination strategies that simultaneously target multiple oncogenic pathways and integrate molecular agents with immune‐based therapies. By simultaneously addressing cell‐intrinsic oncogenic mechanisms and microenvironmental immune barriers, these approaches may achieve deeper and more durable remissions while remaining accessible to CAR T–ineligible patients. As translational research continues to refine molecular subtyping and identify actionable biomarkers, future treatment paradigms in PR‐DLBCL will likely revolve around tailored combinations of cellular therapies, bispecific antibodies, and targeted agents, selected according to the genetic and immunological architecture of each patient's lymphoma [53].

## 6 Conclusions

PR‐DLBCL remains one of the most challenging in contemporary lymphoma management, characterized by intrinsic chemoresistance, aggressive clinical behavior, and a high risk of early mortality [1]. CAR T‐cell therapy has transformed outcomes for many patients in this setting, yet a substantial proportion are unable to receive it due to clinical instability, logistical barriers, transient organ dysfunction, or system‐level constraints. For these individuals, classified as unsuitable rather than ineligible, the therapeutic window is narrow and effective alternatives must be initiated promptly to prevent irreversible clinical decline [7].

Over the past decade, the treatment landscape has expanded considerably. Novel antibody–drug conjugates, bispecific antibodies, immunomodulatory approaches, and targeted agents have begun to fill the therapeutic gap for patients unable to proceed to CAR T‐cell therapy. These innovations allow for immediate disease control, outpatient feasibility, and improved tolerability, offering meaningful responses even in biologically adverse disease. Bispecific CD20 × CD3 antibodies, in particular, have emerged as agents capable of inducing rapid cytotoxicity without the delays associated with autologous cell manufacturing. Similarly, tafasitamab–lenalidomide, polatuzumab‐based combinations, and loncastuximab tesirine provide mechanistically diverse and clinically actionable strategies tailored to the needs of physiologically or logistically constrained patients.

At the same time, the landscape of cellular therapy is evolving. Next‐generation CAR‐T constructs with reduced toxicity profiles, rapid or on‐site manufacturing technologies, and allogeneic CAR‐T or CAR‐NK platforms may soon expand access to patients who are not immediately ready. Integration of bispecific antibodies with targeted therapies and precision‐based combinations holds further promise, particularly as molecular stratification allows increasingly individualized treatment decisions [28].

Improving outcomes for CAR‐T–unsuitable patients require a multipronged approach: early recognition of reversible barriers, timely deployment of effective non‐CAR‐T therapies, expansion of manufacturing and treatment capacity, and continued refinement of biologically informed therapeutic strategies [14]. As the therapeutic ecosystem evolves, the goal will be to convert temporary unsuitability into renewed eligibility whenever possible, while ensuring that patients who remain unable to receive CAR T‐cell therapy still benefit from potent, durable, and accessible treatment options [52]. In this rapidly advancing field, sustained clinical trial participation and robust translational research will be essential to close the survival gap for this high‐risk population.

## Author Contributions

**Santino Caserta**, **Enrica Antonia Martino**, **Mamdouh Skafi**, **Fortunato Morabito**, and **Massimo Gentile:** conceptualization. **Enrica Antonia Martino**, **Francesco Mendicino**, **Ernesto Vigna**, **Antonella Bruzzese**, and **Fortunato Morabito:** methodology. **Enrica Antonia Martino**, **Santino Caserta**, **Fortunato Morabito**, **Massimo Gentile**, and **Nicola Amodio:** writing – original draft preparation. **Enrica Antonia Martino**, **Santino Caserta**, **Mamdouh Skafi**, **Fortunato Morabito**, **Massimo Gentile:** writing, review, and editing. All authors have read and agreed to the published version of the manuscript.

## Funding

The authors have nothing to report.

## Conflicts of Interest

The authors declare no conflicts of interest.

## Data Availability

Data sharing not applicable to this article as no datasets were generated or analysed during the current study.
